# Closing gaps through innovation in the tuberculosis pathology value chain

**DOI:** 10.1016/j.jctube.2026.100623

**Published:** 2026-06-09

**Authors:** Anura David, Lesley Scott, Pedro da Silva, Wendy Stevens

**Affiliations:** a Wits Diagnostics Innovation Hub, Health Sciences Research Office, Faculty of Health Sciences, University of the Witwatersrand, Johannesburg, South Africa; b National Priority Programmes, National Health Laboratory Services, Johannesburg, South Africa

**Keywords:** Tuberculosis, molecular diagnostics, Pathology value chain, additional specimen types, Point-of-care testing

## Abstract

Despite being preventable and curable, tuberculosis (TB) remains the leading cause of death from a single infectious agent globally. In 2024, an estimated 10.7 million people developed TB, yet only 8.3 million were diagnosed, reflecting significant diagnostic gaps. These “missing millions” underscore systemic weaknesses within the TB pathology value chain, defined here as the sequence of processes from patient identification and specimen collection through testing, result reporting, linkage to treatment and monitoring. This review highlights key challenges and opportunities across each step of this chain, with a focus on diagnostic bottlenecks and innovations to improve accessibility, efficiency, and patient-centred care. Sputum remains the primary diagnostic specimen, but it presents barriers for individuals unable to expectorate, such as children and people with HIV. Additional specimens, including stool (for adults), urine (for molecular testing), blood, breath, saliva and oral rinse, show promise, but require further validation. Laboratory constraints, particularly in transport logistics and result turnaround times, contribute to diagnostic delays and patient attrition. While molecular tests and next-generation sequencing have improved TB detection and drug resistance profiling, their decentralization remains limited by infrastructural and financial barriers. Digital tools for result reporting and patient tracking show promise but need broader integration. Near point-of-care technologies and novel diagnostics tailored to regional needs can close critical gaps, reduce loss to follow-up, and support earlier treatment initiation. Ultimately, strengthening the TB diagnostic value chain requires coordinated investments, innovative technologies, and policy frameworks that support equitable access to timely diagnosis and care, particularly in high-burden settings.

## 1 Introduction

Tuberculosis (TB) remains a major global health challenge, despite being both preventable and curable. In 2024, TB remained the leading cause of death from a single infectious agent, accounting for ∼1.23 million deaths worldwide, including approximately 1.1 million deaths among HIV-negative individuals and around 150,000 among people with HIV (PHIV) [1].

Globally, an estimated 10.7 million people developed TB in 2024, of whom 8.3 million were diagnosed and reported, leaving ∼2.4 million individuals who were “missing” from routine health systems. [1]. This gap is due to under-reporting and under-diagnosis due to limited accessibility to healthcare services, insufficient human resources and poor linkages between private providers and national authorities [2].

Drug-resistant TB (DR-TB) poses an additional threat to TB care and prevention. Worldwide in 2019, close to 500,000 people developed rifampicin-resistant TB (RR-TB) of which 78% had multidrug-resistant TB (MDR-TB) [3]. Globally in 2023, 175,923 people were diagnosed and treated for MDR/RR-TB; representing 44% of the 400,000 people estimated to have developed MDR/RR-TB.

As of 2024, the World Health Organization (WHO) continues to identify 30 high TB burden countries, including South Africa (SA), which collectively account for approximately 87% of global TB cases annually [4]. This statistic underscores the concentration of TB incidence within a limited number of countries, highlighting the critical need for targeted interventions in these regions.

Improving the TB diagnostic and treatment cascade is critical to reducing morbidity and mortality and achieving the End TB Strategy (https://www.who.int/teams/global-tuberculosis-programme/the-end-tb-strategy) targets. Effective TB control depends on early detection, prompt initiation of appropriate therapy, and sustained patient engagement throughout care. These goals require a well-functioning pathology value chain that can operate effectively across diverse healthcare settings.

This review examines key weaknesses and opportunities within the TB pathology value chain, with a focus on diagnostic bottlenecks and innovations that may improve efficiency, accessibility, and equity. It highlights current technologies, implementation challenges, and emerging solutions that support more responsive and patient-centered approaches to TB detection and care.

## 2 TB pathology value chain

**Fig. 1:**
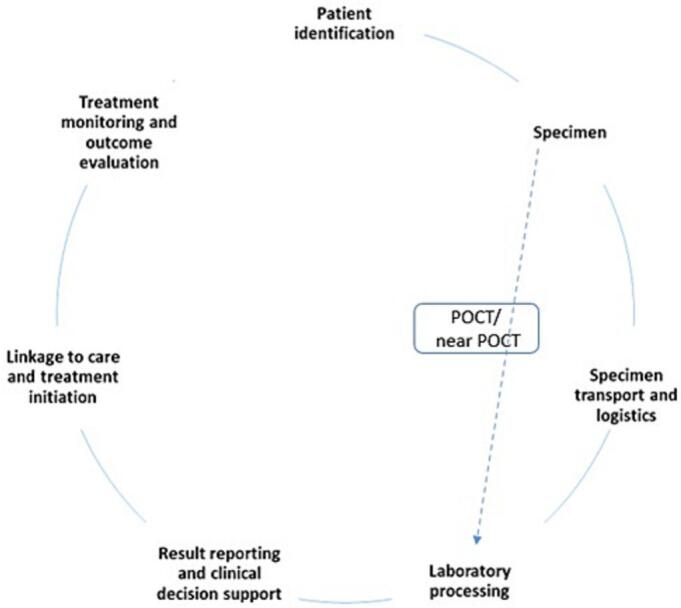
The TB Pathology Value Chain: Key Steps in Diagnosis and Patient Management. This diagram illustrates the TB pathology value chain, highlighting the essential steps involved in diagnosing and managing tuberculosis (TB). A dotted line indicates the potential for point-of-care testing (POCT) to streamline early diagnosis and reduce delays in the chain.

The TB pathology value chain refers to the end-to-end process of TB diagnosis and management, encompassing all steps from specimen collection to treatment monitoring (Fig. 1). It ensures an efficient and patient-centered approach to TB diagnosis and care. The key components include patient identification, specimen collection, specimen transport and logistics, specimen processing, result reporting and clinical decision support, linkage to care and treatment initiation and treatment monitoring and outcome evaluation. Table 1 summarizes each step of the TB pathology value chain, outlining typical challenges and opportunities for improvement across the continuum of diagnostic care.

**Table 1:** Challenges and Innovations in the TB pathology value chain.

Step	Challenges	Opportunities for Improvement
Patient Identification	Passive case finding	Active case finding, digital chest x-rays (dCXR) and computer aided diagnosis (CAD)
Specimen	Difficult to expectorate sputum in paucibacillary disease, invasiveness of specimen types (gastric aspirates and Bronchoalveolar Lavage Fluid)	Use of additional, non-invasive and easy-to-collect specimen types
Specimen Transport & Logistics	Delays in specimen receipt at laboratory, temperature sensitivity, rejected specimens	Improved packaging, more stable specimens
Laboratory Processing	Delayed or inaccurate testing, poor infrastructure, diagnostics with performance limitations, high costs	Molecular diagnostics, decentralization
Result Reporting and clinical decision support	Data entry errors, delay in communication	Digital systems, integration with clinical platforms
Linkage to Care and treatment initiation	Loss to follow-up, poor referral systems, stigma	Near point-of-care tests, mobile clinics, community engagement
Treatment Monitoring and outcome evaluation	Lack of real-time tools, adherence uncertainty, Incomplete data, delayed evaluation, poor feedback to providers	Electronic monitoring, digital adherence technologies, Real-time dashboards, improved reporting mechanisms

This table outlines key challenges across the TB pathology value chain and identifies opportunities to improve diagnostic efficiency, system performance, and patient outcomes.

Analysis of the South African TB care cascade in 2017, as reported by Naidoo et al. [5] revealed patient losses at every stage, with large gaps occurring during the diagnostic phase (13%) and at the point of successful treatment completion (17%). This finding underscores the importance of early and accurate TB detection, as all subsequent steps in the pathology value chain depend on timely diagnosis. Without it, patients may remain untreated, transmission continues, and health outcomes deteriorate. A ten-year analysis of the South African molecular diagnostic programme demonstrated that molecular diagnostics helped avert approximately 4.3 million disability-adjusted life years (DALYs) and generated a return on investment of nearly 14 to 1. This evidence illustrates that strengthening the diagnostic step of the pathology value chain not only improves individual patient outcomes but also yields substantial public health and economic benefits [6].

### 2.1 Patient identification

From a pathology value chain perspective, patient identification represents the critical entry point into diagnostic systems. Traditionally, TB diagnosis relies on passive case finding, whereby individuals experiencing symptoms voluntarily present to healthcare facilities. Those reporting any TB-related symptoms are referred for further evaluation. However, this pathway is influenced by a range of barriers that prevent individuals from being identified and entering the diagnostic cascade. At the patient level, challenges such as time off from work, fear of disclosure, lack of transportation, limited awareness, stigma, and delays in health-seeking behavior reduce presentation to care. At the system level, constraints including insufficient access to diagnostics, overburdened facilities, and weak referral pathways further limit case detection. In addition, structural factors such as poverty, geographic barriers, and inequitable access to healthcare services exacerbate these gaps. Even among those who do access care, the WHO four-symptom screen (W4SS) is insufficient as a standalone screening tool, due to reduced sensitivity in key populations such as individuals on antiretroviral therapy and low specificity [7]. As a result, many individuals who should be investigated for TB are not identified and never enter the diagnostic pathway.

Over the past few years, active case finding (ACF) for TB has gained momentum, driven by renewed global emphasis on systematic screening to recover declines in case detection during the COVID-19 pandemic [4]. ACF involves screening individuals in communities, high-risk groups, or congregate settings (e.g., prisons, mines, urban informal settlements), regardless of symptoms, using approaches such as door-to-door screening, mobile clinics, contact investigation, or targeted campaigns. A key tool supporting ACF is digital chest X-ray (dCXR), with or without computer-aided detection (CAD), which enables rapid identification of individuals with abnormalities suggestive of TB and prioritization for confirmatory testing [8]. However, adoption remains constrained by cost, infrastructure, and implementation challenges [9]. Although evidence on the effectiveness of ACF is mixed [10], it has the potential to accelerate TB elimination by shortening time to diagnosis, reducing transmission, and identifying otherwise missed cases. Its impact, however, depends on strong health system support and local context. Accordingly, WHO recommends targeted screening of high-risk populations, supported by robust diagnostic capacity and effective linkage to care [11] as a complement to passive case finding. Strengthening patient identification therefore requires not only improved screening tools but also the capacity to translate increased case detection into timely diagnosis and treatment, emphasising the need for integrated and context-appropriate implementation strategies.

### 2.2 Specimen

For pulmonary TB, sputum is the primary diagnostic specimen, with both adequate quality and a minimum volume of 2 mL required to ensure accurate testing [12]. However, recent studies indicate that quality assessment criteria may need to be tailored to specific regions or countries, as test performance can vary across settings [13] especially in the era of molecular testing [14]. A major limitation of sputum-based diagnosis is that some individuals, particularly those with paucibacillary disease may be unable to produce sputum. When sputum cannot be easily expectorated, sputum induction is an alternative for collecting airway secretions. While effective, this method carries a risk of bronchoconstriction and requires lung function monitoring before and during the procedure [15]. Additionally, sputum induction is not feasible in community-based ACF programs. A study conducted in SA's Eastern Cape province found that approximately 83% of household contacts were unable to produce sputum [16], highlighting a significant barrier to TB testing in such settings. Other specimen types recommended for pulmonary TB diagnosis include gastric aspirates and bronchoalveolar lavage fluid (BALF) which are more invasive specimen types.

The need to improve access to diagnostic testing has driven the search for non-sputum specimens, particularly for use with molecular platforms, with promising results.

WHO recommends stool as an alternative specimen type for *Mycobacterium tuberculosis* complex (MTBC) detection in children [17] providing a useful option for those unable to produce sputum despite variable performance in paucibacillary disease [18]. Simplified stool processing methods have also been developed to improve the feasibility of implementation [19]. A study in adults, including PHIV, show that stool Xpert MTB/RIF Ultra (Ultra) has high specificity but relatively low sensitivity, while contributing additional diagnostic yield when used alongside existing diagnostics [20]. These findings suggest that stool may serve as a useful adjunctive specimen in adults, although further validation is required.

The WHO recommends the Alere Determine™ TB LAM Ag (Alere LAM) assay to support TB diagnosis in PHIV, particularly those who are seriously ill, reflecting the utility of urine-based testing [17]. Although the sensitivity of urine for molecular tests such as Ultra is low [21], the combined use of urine-based molecular assays and Alere LAM alongside sputum testing has been shown to enhance diagnostic yield and improve patient outcomes [22], [23].

Blood is not recommended by WHO as a specimen type for diagnosis of active TB disease, however an assay designed by Cepheid which uses finger-stick blood and assesses TB infection through the host immune response is in process of evaluation [24]. The assays' utility in the fight against TB is yet to be determined.

Breath tests for TB diagnosis have also shown promise as an alternative specimen type but evaluation in larger studies across diverse populations is limited [25]. Face mask sampling involving capture of MTBC from breath using a mask is also being investigated [26] but like other alternative non-sputum specimens, requires optimisation to improve sensitivity.

Saliva, previously considered suboptimal for TB diagnosis, has now been shown to be a feasible specimen for the detection of MTBC using Ultra and has demonstrated promising diagnostic performance [27].

Oral rinse is another specimen that has shown potential for TB diagnosis when paired with molecular testing [28]. However, data is limited, diagnostic performance is variable and generally lower than sputum.

Recent developments have introduced swab-based sampling approaches, including swabs dipped in sputum and tongue swabs (TSs) for TB diagnosis. Both swab types are recommended by the WHO, with TSs only being recommended when sputum cannot be obtained, particularly for use in conjunction with near point-of-care (NPOC) molecular nucleic acid amplification tests (NAATs) [29]. Studies report TS sensitivity of 75%–86% and sputum sensitivity of 86%–94%, both with high specificity, with sputum performance comparable to Xpert Ultra [30], [31], [32]. Importantly, TSs and other non-sputum specimen types are primarily used in conjunction with molecular diagnostics rather than conventional culture-based methods, and their utility is therefore closely linked to the availability of such platforms. In settings where smear microscopy remains the only available diagnostic tool, swabs paired with a NPOC assay may offer a valuable alternative [33].

As mentioned, performance data on additional specimen types, demonstrate lower sensitivity compared with sputum. However, Broger et al. [34] illustrates that tests with limited sensitivity can diagnose more people with TB if they allow increased diagnostic yield. Non-sputum specimens improve accessibility for non-productive individuals and offer additional advantages, including easier collection, potential self-collection, less invasive collection and reduced risk of aerosolization. Alternative specimen types such as stool (in adults) and urine for molecular testing have shown promising evidence for TB diagnosis, whereas others, including blood, breath, saliva, and oral rinse, remain under investigation and require further validation and protocol optimisation. The value of these alternative specimens lies not only in their diagnostic performance but also in their potential to improve access to testing across the pathology value chain, particularly in resource-limited settings. As demonstrated with TSs, robust evidence is needed to establish performance and guide policy, highlighting the importance of continued research to support their adoption.

### 2.3 Specimen transport and logistics

If onsite TB testing is unavailable, specimens must be transported to a testing facility. Sputum storage and transportation at refrigerated temperatures (2–8 °C) is crucial for maintaining specimen integrity for diagnosis [12]. However, this requirement poses significant challenges in settings, where access to cold-chain storage, reliable electricity, and efficient transport networks may be limited. These logistical constraints also have financial implications, increasing the overall cost of TB diagnostics.

Another major concern is delayed diagnosis due to long transit times from collection facility to testing facility, which can lead to disease progression and continued transmission within communities. These constraints highlight how inefficiencies in specimen transport can undermine the entire diagnostic pathway, emphasising the need for system-level improvements to ensure timely and reliable testing. Additionally, specimen rejection is a frequent issue, with the most common reasons being insufficient sputum volume and leakage from improperly sealed containers. Data from the Central Data Warehouse (National Priority Programmes, National Health Laboratory Service, South Africa) indicate that approximately 100,000 sputum specimens submitted for TB testing in 2024 were rejected due to insufficient volume (https://cdwmicrostrategy.nhls.ac.za).

One approach to overcoming these challenges, investigated by Doerfler et al. [35] and Zerbini et al. [36] is the use of re-engineered sputum collection cups designed to minimize leakage and facilitate laboratory processing. Another possible solution is the use of additional specimen types, such as TSs, which are less prone to leakage and can potentially be transported at room temperature. A third strategy could involve collecting two sputum specimens upfront, a method shown to decrease diagnostic turnaround time. With this approach, ∼93% of unsuccessful tests were repeated within five days of initial result, compared to just 52.5% with a single-sputum collection method [37]. However, the additional costs associated with increased specimen collection, transport, storage, and waste generation may limit the feasibility of this approach in routine settings. Improved collection devices and additional specimen types may therefore be more feasible, scalable options.

### 2.4 Laboratory processing and diagnosis

Within the pathology value chain, laboratory processing constitutes a central step where diagnostic delays frequently occur. The introduction of NAATs such as the Xpert MTB/RIF (Xpert) and later, the Ultra, significantly improved the speed and efficiency of TB diagnosis. Both tests are endorsed by WHO as the initial diagnostic test in individuals with presumed DR-TB or HIV-associated TB [38], [39]. Although originally promoted as point-of-care (POC) technologies to reduce reliance on centralized laboratory infrastructure and specimen transport, implementation has not consistently achieved this goal. In many low- and middle-income countries (LMICs), these platforms have predominantly been deployed at higher-level facilities, meaning that specimen transport and associated delays remain barriers within the diagnostic pathway [40]. Furthermore, WHO has reclassified this technology in a new class labelled as “low complexity” since operational and infrastructural requirements prevent it from being a fully decentralized or field-based test [41].

Despite the advantages of molecular diagnostics, their uptake has been variable, with implementation constrained by infrastructure limitations, high costs, requirements for skilled personnel, and data management challenges [42]. In many resource-limited settings, smear microscopy therefore remains the only widely sustainable diagnostic due to its low cost and minimal infrastructure requirements, despite its lower sensitivity [43]. This reflects a persistent gap between technological innovation and practical scalability within the TB diagnostic landscape.

For the diagnosis of DR-TB, WHO recommends use of targeted next generation sequencing (tNGS) [44], which enables comprehensive detection of resistance, including to newer agents such as bedaquiline, clofazimine, and linezolid. In addition, bioinformatics tools such as MAGMA, TB-Profiler, and Mykrobe have improved the accessibility of sequencing analysis for clinical use [45], [46]. However, implementation of sequencing in routine settings remains challenging, as it requires substantial infrastructure, technical expertise, and reliable supply chains, and its diagnostic performance declines in specimens with low bacterial load [47]. These limitations restrict its applicability in decentralized or resource-constrained settings.

Culture remains the reference standard for TB diagnosis and is essential for phenotypic drug susceptibility testing (pDST). However, culture-based workflows introduce additional delays and complexity within the diagnostic pathway. In TB culture laboratories, a MGIT contamination rate of 3–8% is considered acceptable [7]. While this level of contamination is manageable from a laboratory perspective, it poses a significant challenge for patients whose TB treatment decisions rely on a pDST result. Attempts to recover bacilli from contaminated cultures, for example through re-decontamination, have been reported to be successful in ∼50% of cultures [31], further prolonging time to diagnosis and treatment initiation. If the culture cannot be salvaged, an additional sputum will be required by the laboratory. Similarly, unsuccessful NAAT results, including errors or invalid outcomes, occur in approximately 2% of tests in large-scale programmes such as SA's national GeneXpert program [48]. These challenges highlight how laboratory processes themselves contribute to diagnostic delays.

The Alere LAM test represents the only WHO-recommended true POC TB diagnostic; however, its use is restricted to certain populations [49]. In addition, the test does not distinguish between MTBC and non-tuberculous mycobacteria [50], limiting its broader applicability.

More broadly, while several emerging technologies aim to address current diagnostic gaps, including cartridge-based molecular assays, sequencing approaches, and host-response biomarkers [51], many remain constrained by similar challenges related to cost, infrastructure, and scalability.

Although many of these newer technologies are designed for sputum, manufacturers have recognized the advantages of testing additional specimen types and many are now developing NPOC assays meant specifically for swab-based specimens. These assays potentially allow decentralized testing and will have a role to play in the fight against TB but, currently, they are able to only detect MTBC and not drug resistance. As a result, these assays are likely to serve primarily as screening tools, with confirmatory testing continuing to rely on centralized sputum-based methods.

Lessons learned from the implementation of GeneXpert highlight that a single assay cannot meet global TB diagnostic needs [52], [53]. Countries, even specific regions within a country, require tailored solutions based on their unique challenges and resources. In high-burden settings, high-throughput centralized testing is essential to support initiatives like ACF. However, using SA as an example, although the country has a high overall TB burden, there is marked spatial variability in both TB positivity and RIF resistance across the country [54], necessitating the adoption of different technologies to better align with regional needs [55]. With the rapid expansion of technologies across specimen types, countries now have far more options to tailor diagnostic strategies to their own epidemiological realities and health system capacities. Implementers can now can draw from a growing toolbox of innovations to overcome local barriers, close diagnostic gaps, and accelerate progress toward equitable and effective TB care. The transformative potential of molecular diagnostics, particularly for rapid detection of disease and drug resistance, will depend on context-appropriate implementation, sustained investment in health systems, and effective integration into routine services. Addressing these requirements can help close the persistent gap between technological innovation and real-world scalability, ensuring that advances are not only technically robust but also operationally feasible and responsive to local health system capacities.

### 2.5 Result reporting and clinical decision support

Once a result is available at the laboratory, these results need to be reported to the healthcare provider for patient management. However, barriers also exist in this step of the value chain. These include delays in reporting due to slow turnaround times or delayed communication; errors in data transmission; poor integration of healthcare systems between the laboratory information systems (LIS) and hospital systems; misinterpretation of results due to unclear or incomplete reports or lack of interpretation guidelines; incorrect patient identifiers or inadequate follow-up of results by healthcare providers [56], [57], [58], [59].

These barriers in the reporting phase can be mitigated through targeted digital health interventions [60] that enhance communication, reduce delays, and improve data accuracy. For instance, short message service (SMS)-based systems have proven effective in accelerating result delivery to healthcare providers, and even directly to patients, particularly in resource-limited settings such as Uganda and SA [55], [61], reducing reliance on manual result retrieval. Furthermore, digital tools such as eLabs (https://mezzanineware.com/digital-productivity-technology/healthcare-technology-solutions/laboratory-improvement-technology/), an electronic laboratory information management and tracking system, enable real-time monitoring of specimens from collection to result dispatch. This platform improves integration between laboratories and clinical care settings by streamlining data transmission, minimizing errors, and enabling better follow-up through traceable patient identifiers and automated alerts. By digitizing these critical steps in the laboratory value chain, such innovations directly address common bottlenecks, including delayed communication and poor system interoperability (53). This highlights the importance of integrated digital health systems that link laboratories and clinical services, ensuring that diagnostic innovations translate into timely clinical decision-making.

### 2.6 Linkage to care and treatment initiation

Even after a laboratory result is generated and received by the healthcare provider, ensuring effective linkage to care remains a critical challenge in the TB pathology value chain. If patients do not return to the facility or cannot be contacted, they are effectively lost to follow-up, resulting in delayed or missed treatment initiation. Several previously-mentioned patient-reported barriers contribute to this issue, including unclear instructions on when to return for results.

Loss to follow-up (LTFU) at this stage is a significant concern, with rates reported between 12.5%–25% [62], [63]. Additional obstacles arise when laboratory results are unsuccessful, requiring patients to return to provide a second specimen and make further visits for results and treatment, thereby increasing the risk of attrition. Communication breakdowns between healthcare facilities and patients, as well as gaps in education among both patients and healthcare workers, further exacerbate these challenges. Moreover, the lack of patient-centred approaches often hinders effective TB treatment and follow-up [64].

To address these barriers, improved communication strategies, such as patient counselling and mobile messaging, can help reduce misunderstandings and alleviate fears. Decentralized testing with same-day result delivery offers a particularly effective solution. True POC and NPOC diagnostics that provide rapid TB, and, in future, drug resistance results can reduce the need for repeat visits, strengthen linkage to care, and enable prompt treatment initiation. These innovations are especially valuable at this vulnerable stage of the pathology value chain, where patient loss can undermine TB control efforts. Ultimately, the impact of improved diagnostics depends on effective linkage to care, emphasising the need for patient-centred approaches that minimize delays and loss to follow-up.

### 2.7 Treatment Monitoring and outcome evaluation

Treatment initiation marks only one phase in the pathology vale chain. Effective treatment monitoring is essential to ensure the successful completion of treatment, prevent the development of drug resistance, support patient adherence, and reduce the spread of the disease. It is a crucial component of TB control programs, as ongoing monitoring ensures that patients are on the correct therapeutic regimen, particularly considering rising DR-TB cases. Recurrent TB has been shown to be relatively common soon after treatment completion, particularly within the first year for RS-TB [65]. Key risk groups, such as men, PHIV, and those in high TB-burden areas, may benefit from intensified follow-up.

In addition to monitoring bacteriological response, there is a need for systematic surveillance of treatment-related adverse drug reactions, particularly in patients who require prolonged regimens. Van der Walt et al. (2013) reported that 6.9% of MDR-TB patients experienced at least one serious adverse drug reaction (SADR), most commonly ototoxicity. While HIV-positive, antiretroviral therapy (ART)-naïve patients did not show increased SADR risk compared to HIV-negative patients, the study highlights the need for careful pharmacovigilance, especially as treatment decentralizes and TB-HIV co-treatment expands [66].

Current monitoring tools, such as culture and smear microscopy, have significant limitations. Both methods are slow, have limited sensitivity, and fail to track drug resistance or assess the effectiveness of treatment in real time. This creates a major gap in the pathology value chain, as timely information about treatment response is critical to guide clinical decisions.

Emerging solutions, such as electronic medication monitors (EMMs), have shown promise in improving treatment adherence and success rates. Studies conducted in China [67] and SA [68] have demonstrated the potential of EMMs to enhance patient compliance and treatment outcomes. However, data on the direct impact of these tools on TB treatment outcomes remains limited.

Currently, treatment success is defined by the completion of therapy [69], but this approach lacks a reliable way to confirm that the disease has been fully eradicated or that drug resistance has not developed. Emerging approaches include molecular assays that quantify mycobacterial load (e.g. RNA-based tests targeting viable bacilli) and host-response biomarkers, which offer more rapid and potentially more accurate measures of treatment response than conventional microscopy and culture, and may improve assessment of treatment success and accelerate the evaluation of new TB regimens [70]. Greater innovation in TB treatment monitoring is needed to improve patient outcomes and support the development of new therapies. Addressing these gaps will require scalable, real-time monitoring tools that can be integrated into routine health systems, enabling more responsive and patient-centred TB care.

## 3 Conclusion

Addressing the challenges within the TB pathology value chain requires a multi-faceted approach that prioritizes accessibility, sustainability, and integration of diagnostic innovations into healthcare systems. While molecular assays and novel specimen types offer transformative potential, their impact is hindered by infrastructural limitations, inconsistent funding, workforce shortages, and fragmented implementation strategies. To overcome these barriers, concerted efforts must focus on strengthening laboratory networks, investing in capacity-building, ensuring reliable supply chains, and fostering policy frameworks that support equitable access to diagnostics. Public-private partnerships, community engagement, and innovative financing mechanisms can further accelerate adoption and scale-up. Ultimately, a patient-centered approach, where diagnostics are brought closer to the POC and seamlessly integrated into clinical workflows, will be key to closing diagnostic gaps and achieving global TB elimination goals.

## CRediT authorship contribution statement

**Anura David:** Writing – review & editing, Writing – original draft, Visualization, Resources, Methodology, Investigation, Formal analysis, Conceptualization. **Lesley Scott:** Writing – review & editing, Supervision, Funding acquisition. **Pedro da Silva:** Writing – review & editing, Supervision, Funding acquisition. **Wendy Stevens:** Writing – review & editing, Supervision, Funding acquisition.

## Ethical statement

Ethics approval was not required for this work as it did not involve human participants, animals, or the use of identifiable personal data.

## Funding

Wendy Stevens, Lesley Scott and Anura David were supported by funding from the 10.13039/100000865Gates Foundation (OPP1171455). Lesley Scott, Pedro da Silva and Wendy Stevens are also supported by 10.13039/100000002National Institutes of Health (R01AI152126). The funders had no role in the collection, analysis, or interpretation of information; in the writing of the

paper; or in the decision to submit the paper for publication.

## Declaration of competing interest

The authors declare that they have no known competing financial interests or personal relationships that could have appeared to influence the work reported in this paper.
